# Osthole attenuates asthma-induced airway epithelial cell apoptosis and inflammation by suppressing TSLP/NF-κB-mediated inhibition of Th2 differentiation

**DOI:** 10.1186/s13223-024-00913-8

**Published:** 2024-09-27

**Authors:** Yanli Li, Yushan Zhou, Liqiong Liu, Yunfeng Yang, Yanhong Liu, Dailing Yan, Juyan Chen, Yi Xiao

**Affiliations:** Department of Respiratory and Critical Care Medicine, Yan’an Hospital of Kunming City, No. 245 Renmin East Road, Kunming, 650051 China

**Keywords:** Bronchial asthma, Osthole, TSLP, NF-κB, Th2 differentiation

## Abstract

**Objective:**

The aim of this study was to investigate the influence of osthole (OS) on asthma-induced airway epithelial cell apoptosis and inflammation by restraining Th2 differentiation through suppressing TSLP/NF-κB.

**Methods:**

An asthma mouse model and an inflammation cell model were constructed with ovalbumin (OVA) and lipopolysaccharide (LPS), respectively. CD4 + T cells were treated with IL-4 to induce Th2 differentiation. Model mice were treated with OS (15,40 mg/kg) for 7 days, and 10 µg/mL OS was added to cell treatment groups. The levels of relevant indices were detected by RT‒qPCR, HE and Masson staining, Western blotting, ELISA and flow cytometry.

**Results:**

In a mouse asthma model, TSLP expression was elevated, and the NF-κB pathway was activated. Therefore, OS could restrain the apoptosis and inflammation of airway epithelial cells. Downstream mechanistic studies revealed that OS can suppress Th2 differentiation by restraining the level of TSLP and NF-κB nuclear translocation, thus facilitating the proliferation of airway epithelial cells, restraining their apoptosis and inflammation, and alleviating airway inflammation in asthmatic mice.

**Conclusion:**

OS can inhibit Th2 differentiation by inhibiting the TSLP and NF-κB pathways, which can reduce the apoptosis and inflammation of airway epithelial cells caused by asthma.

**Supplementary Information:**

The online version contains supplementary material available at 10.1186/s13223-024-00913-8.

## Introduction

Bronchial asthma is a chronic inflammatory disease characterized by reversible airway obstruction, significant airway hyperresponsiveness, peribronchial inflammation, and airway remodeling [1]. It is caused by immune dysfunction, and the instability of Th1/Th2 CD4^+^ Th cell subsets is deemed to be involved in the immune pathogenesis of asthma [2]. At present, there is no radical cure for bronchial asthma, and it is characterized by a sudden onset, long process and liable relapse [2]. It incurs tremendous economic encumbrance to patients and society. Hence, there is an urgent need to develop new anti-bronchial asthma treatments.

Osthole (7-methoxy-8-isopentenylcoumarin, OS) is an artificial coumarin from *Cnidium monnieri* fruit. The pharmacological activities of OS include antioxidant, anti-inflammatory, anti-allergic, and antidiabetic effects [3]. OS plays an important role in the regulation of many diseases; for example, Chen [4] reported that OS can reduce lipopolysaccharide-induced acute lung injury in mice. Li [3] showed that OS can also restrain inflammatory cell infiltration, collagen sedimentation and proinflammatory cytokines in asthmatic mice, thus playing a therapeutic role. In this study, we explored the mechanism of OS in the treatment of asthma and applied a novel theoretical reference for asthma therapy.

Thymic stromal lymphopoietin (TSLP) is an epithelium-derived immune factor that regulates the inflammatory response of helper T (Th) cells [5]. TSLP was found to play a pivotal pathogenic role by serving as an upstream catalyst of cellular and molecular pathways involved in airway inflammation in type 2 (T2 high) patients [6]. Moreover, OS can notably restrain atopic dermatitis by directly suppressing the level of TLSP in keratinocytes [7]. Therefore, exploring the molecular mechanism by which OS regulates bronchial asthma through TSLP could reveal new therapeutic targets. In addition, TSLP production is interrelated with the revitalization of the NF-κB pathway [8]. NF-κB plays a critical role in the maintenance and control of acute inflammation, and many proinflammatory cytokine genes are regulated by NF-κB [9]. Schuliga [10] confirmed that NF-κB plays a central role in airway inflammation in asthma and chronic obstructive pulmonary disease. The above studies confirmed that NF-κB plays an important role in the occurrence and development of asthma. This study investigated the mechanism of NF-κB in asthma.

Helper T (Th) cells play a decisive role in immune dysfunction and are conducive to the progression of asthma [11]. Th cells are split into 4 subtypes: interferon (IFN)-γ-excreting Th1 cells, interleukin (IL)-4-excreting Th2 cells, IL-17-generating Th17 cells, and CD4 CD25 Foxp3 regulatory T cells (Tregs) [12]. It has been reported that the instability of Th1/Th2 and Treg/Th17 cells may be pivotal factors in the severity of asthma [13]. Bae [14] reported that cytokines such as IL-4, IL-5 and IL-13 are generated by Th2 cells and are the primary factors involved in the onset of asthma-related inflammation. TSLP can induce Th2 differentiation and initiate the Th2 immune response [15]. Moreover, the NF-κB pathway can regulate the differentiation of Th2 cells and improve the imbalance of Th1/Th2 cells [16]. These results may serve as a reference for investigating the role of Th2 cells in asthma and may represent a novel direction for the study of the latent mechanism by which TSLP/NF-κB regulates Th2 differentiation in asthma.

Therefore, this study investigated the effect of OS on asthma inflammation through the suppression of Th2 differentiation and the use of an academic reference for clinical remedies.

## Materials and methods

### Construction of a mouse asthma model

Thirty female BALB/c mice (18–22 g) aged 6–8 weeks were obtained from the Animal Experimental Center of Kunming Medical University. Mice were maintained under specific pathogen-free conditions with free access to food and water. The mice were randomly divided into 5 groups: the control group, ovalbumin (OVA) group, OVA + OS group (15 mg/kg), OVA + OS group (40 mg/kg) and OVA + dexamethasone (Dex) group (1 mg/kg), with 6 mice in each group. The OVA mouse asthma model was established according to the methods of Li [3] et al. OVA (100 µg) and aluminum hydroxide (2 mg) were dissolved in 200 µL of saline (0.9%), injected intraperitoneally into mice on days 0, 7, and 14, and then intranasally instilled with 50 µL of OVA (20 mg/mL) from days 15 to 28. The control mice were injected intraperitoneally with an equal volume of 0.9% saline. OS (15,40 mg/kg) was administered 1 h before OVA treatment from Days 22 to 28. Dex (1 mg/kg) was injected intraperitoneally 1 h before OVA treatment from Days 15 to 28. The control mice were injected with equal amounts of 0.9% saline for 14 consecutive days. The mice were euthanized by isoflurane anesthesia 24 h after the last treatment and examined for pathophysiological and immunological features of asthma.

### Cell culture and modeling

Human bronchial epithelial (16HBE) cells were obtained from Otwo Biotechnology Co., Ltd. (HTX2577, Shenzhen, China). CD4 + T cells were isolated from normal mouse blood specimens with a CD4 + T-cell kit (Miltenyi Biotec, Germany). All cells were cultured in complete RPMI-1640 medium (Gibco, USA) supplemented with 1% penicillin/streptomycin (Gibco) and 10% FBS (Gibco). The incubator conditions were 37 °C, 5% CO_2_, microscopic observation of cell morphology, routine substrate changes and passage. 16HBE cells were treated with lipopolysaccharide (LPS, 1 mg/L) at 37 °C for 24 h to construct an inflammatory model, CD4 + T cells were treated with 200 U/mL IL-4 to induce Th2 differentiation, OE-TSLP was transfected into CD4 + T cells with Lipofectamine 2000 reagent (Invitrogen, Grand Island, NY, USA), and 10 µg/mL OS was added to all the above cell treatment groups.

### HE staining

The left lung tissue of the mice was removed, dehydrated and embedded in paraffin, dewaxed and hydrated in gradient ethanol, dyed with hematoxylin water, stained with acidic water and ammonia water, and flushed with running water; 70% and 90% ethanol were used for decolorization, respectively; the samples were dyed with alcohol-eosin staining solution and dried at room temperature; xylene was used for clearing, and resin was used for sealing. The cells were observed and photographed under a microscope (Eclipse 80i, Nikon, Japan).

### Masson staining

The paraffin sections were dewaxed and hydrated with gradient ethanol, dyed with Regaud hematoxylin for 5–10 min, flushed thoroughly with water, and immersed in Masson ponceau acid fuchsin for 5–10 min and 2% aqueous glacial acetic acid. The samples were differentiated with 1% aqueous phosphomolybdic acid for 3–5 min, dyed with aniline blue or light green for 5 min without flushing, and dipped in 0.2% glacial acetic acid (aqueous) for a while and was encapsulated with 95% alcohol, xylene (transparent), absolute alcohol, and neutral gum. A microscope (Eclipse 80i, Nikon, Japan) was used to observe the samples and take pictures.

### ELISA test

Mouse serum, bronchoalveolar lavage fluid (BALF) and cell-free culture supernatant were collected after treatment according to the specifications of the ELISA kit (Abcam, UK). The levels of IgE; the Th2 cytokines IL-4, IL-5, and IL-13; the Th1 cytokines IFN-γ and IL-2 in the BALF; and the cellular inflammatory cytokines IL-6, TNF-α and IL-1β were measured with a microplate spectrophotometer (Bio-Tek, USA) at an optical density of 450 nm.

### RT-qPCR

Total RNA was extracted from cells and tissues with TRIzol reagent (Solarbio, Beijing, China). RNA was reverse-transcribed into cDNA by a one-step reverse transcription kit (Takara, Japan). Real-time fluorescence quantitative PCR was performed using a SYBR Green real-time fluorescence quantitative PCR kit (Qiagen, Germany) with GAPDH as an internal control. The thermal cycler conditions for qPCR were 95 °C for 2 min, followed by 40 cycles of 95 °C for 15 s, 60 °C for 20 s. The detailed primer sequences are shown in Table 1, and differential gene expression was analyzed by the 2^−ΔΔCt^ method.

**Table 1:** Primer sequences

Target	Sequence(F: Forward primer; *R*:Reversed primer)(5´-3´)
DNMT1 (16HBE)	F: ACGGTGCGAGACACGATG
	R: TGGTGACGGTTGTGCTGA
TSLP (16HBE)	F: CAACTTGTAGGGCTGGTG
	R: TTGCCTGAGTAGCATTTATC
DNMT1 (mice)	F: CTGGCTAAAGTCAAGTCCC
	R: GGTTCCCGCTGTTACCTC
TSLP (mice)	F: TCAGGAGCCTCTTCATCC
	R: ACTTCTTGTGCCATTTCC
T-bet	F: GGACCCAACTGTCAACTGC
	R: TGTCGCCACTGGAAGGAT
Gata-3	F: GCCATTCGTACATGGAAGC
	R: CGGAGGGTAAACGGACAGAG
GAPDH	F: GGGAAACTGTGGCGTGAT
	R: AAAGGTGGAGGAGTGGGT

### Western blot analysis

Proteins were extracted from cells and lung tissues with radio immunoprecipitation assay (RIPA) buffer (Sigma‒Aldrich, USA) containing 1% protease inhibitors. The protein concentration was determined according to the instructions of the BCA assay kit (Thermo Scientific, USA). Total proteins were separated by SDS‒PAGE, the separated proteins were transferred to PVDF (Millipore, USA) membranes, which were blocked with 5% nonfat milk for 1 h at room temperature. Diluted primary antibodies against DNMT1 (1:1000, ab136360), TSLP (1:1000, ab188766), NF-κB p65 (1:100, ab32536), IκBα (l:10000, ab32518), and p-IκBα (1:1000, ab133462) were added and incubated overnight at 4 °C. Next, the membrane was incubated with secondary antibody (1:2000, ab97051) for 1 h at room temperature and stained with an enhanced chemiluminescence (ECL) kit (Millipore, USA). Ultimately, the bands were semiquantitatively analyzed by ImageJ.

### CCK-8 detection of cell proliferation

16HBE cells (5 × 10^3^/well) were seeded in 96-well plates and cultured at 37 °C in a 5% CO_2_ incubator for 24 h, after which the cells were divided into groups. After treatment, 10 µL of CCK-8 reagent (Sigma‒Aldrich, USA) was added to each well and incubated for 2 h. The absorbance was measured at 450 nm by a microplate reader (ELX800, Bio-Tek, USA).

### Detection of apoptosis by flow cytometry

The cells were collected, flushed twice with PBS, and resuspended in 200 µL of PBS. The percentage of apoptotic cells was determined according to the manufacturer’s instructions. Then, 5 µL of PI and 5 µL of Annexin V-FITC (Absin, China) were added to each tube, and the cells were incubated in the dark for 15 min. Flow cytometry (BD FACSCalibur, USA) was used to detect apoptosis.

### Detection of lactate dehydrogenase (LDH) content by a kit

16HBE cells were collected into a centrifuge tube, and the supernatant was discarded after centrifugation. The cells were lysed by ultrasound according to the ratio of the number of cells (10^4^) to the volume of the extract (mL) (500:1), and the cells were centrifuged at 8000 × g for 10 min at 4°C. The supernatant was collected on ice for analysis. According to the instructions of the LDH kit (BC0685, Solarbio), the samples were mixed with the reagent, and then the LDH content was measured.

### Detection of NF-κB nuclear translocation by immunofluorescence

Cells were inoculated in 24-well plates (2 × 10^4^/well). After 24 h, the cells were flushed twice with PBS, fixed with 4% paraformaldehyde for 30 min, incubated with 0.5% Triton X-100 for 10 min, and blocked with bovine serum albumin for 1 h. Subsequently, the cells were incubated with a primary antibody against NF-κB (1:100, ab32536, Abcam) overnight at 4°C. The next day, the cells were incubated with the corresponding secondary antibody for 1 h and stained with DAPI. Ultimately, the stained cells were observed under a fluorescence microscope (Eclipse 80i, Nikon, Japan), and images were taken.

### Detection of Th1 and Th2 cells by flow cytometry

The cells were collected and suspended in RPMI-1640 medium supplemented with 10% FBS. After 72 h of culture, the indicated cells were collected and suspended. The cell suspensions were incubated with phorbol myristate acetate (PMA, 25 ng/ml), ionomycin (1 µg/ml), and golgistop for 4 h at 37 °C. Th1 and Th2 cell subsets were analyzed for the surface antigen IFN-γ (1:300, abcam) and the intracellular cytokine IL-4 (1:100, abcam).

### Statistical analysis

GraphPad Prism 8.0 (GraphPad, USA) was used to analyze the experimental data and plot the graphics. All data were expressed as means ± SD. All experiments were replicated at least 3 times. One-way ANOVA and t tests were used for statistical analysis. *p* < 0.05 was considered to indicate statistical significance.

## Results

### Upregulation of TSLP and activation of the NF-κB pathway in asthmatic mice

**Fig. 1:**
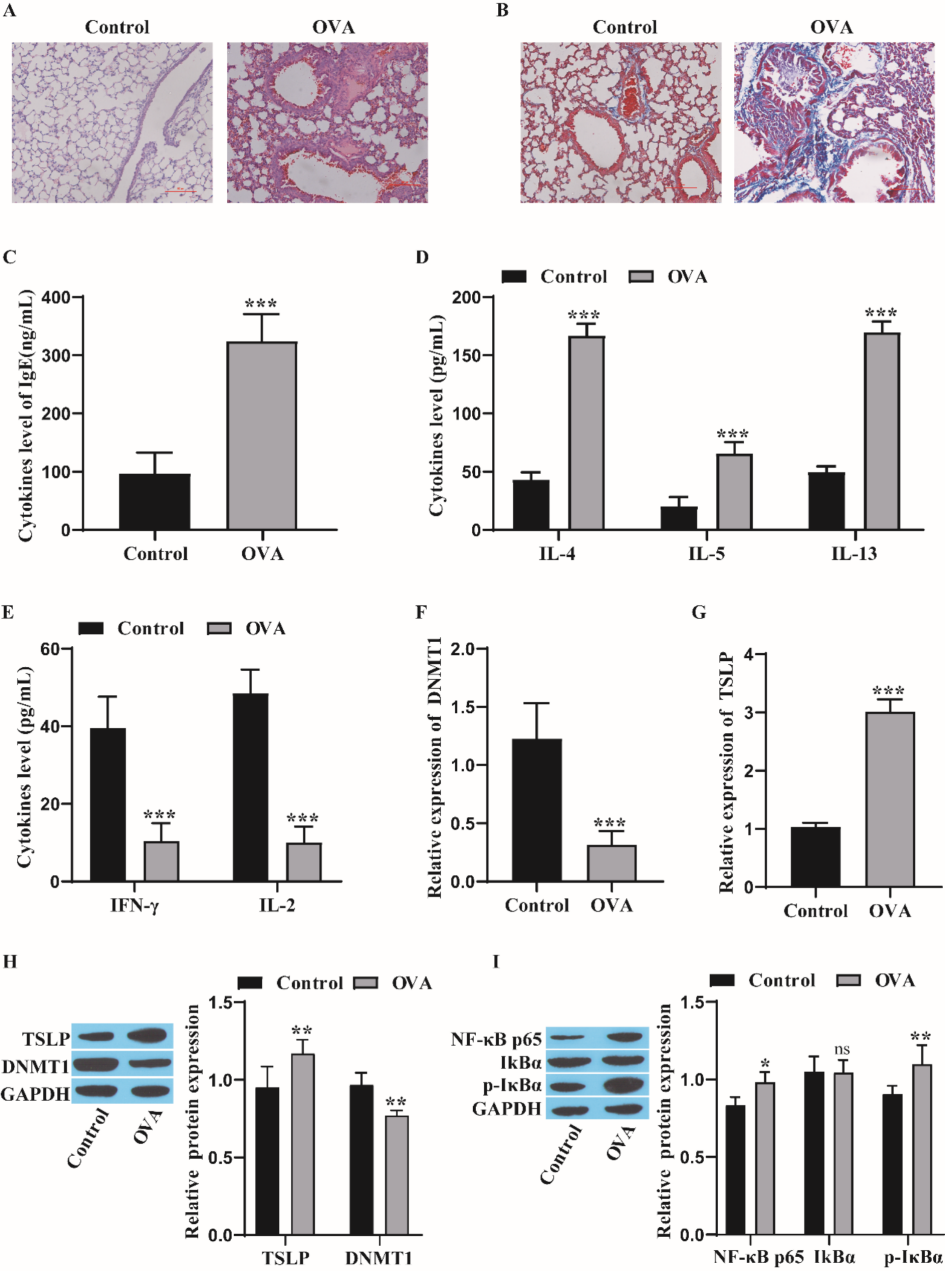
TSLP expression is upregulated and the NF-κB pathway is activated in asthmatic mice. **A**: HE staining was used for observing pathological alterations in lung tissue; scale bar = 100 μm. **B**: Masson staining was used to detect the deposition of collagen in lung tissue; scale bar = 100 μm. **C**: The IgE levels were detected by ELISA; **D**: ELISA was used to measure the levels of Th2 cytokines; **E**: ELISA was used to detect the levels of the Th1 cytokines IFN-γ and IL-2 in the BALF of mice; **F**: The DNMT1 levels were detected by RT‒qPCR; **G**: The TSLP levels were detected by RT‒qPCR; **H**: Western blot was used for measuring the protein levels of DNMT1 and TSLP; **I**: Western blot was used for measuring the levels of NF‒κB pathway proteins. Animals were divided into 2 groups: control group (*n* = 5), OVA (model) group (*n* = 5). The experiments were repeated five times. ^*^P < 0.05, ^**^P < 0.01, ^***^P < 0.001 vs. Control, ns: *P* > 0.05. OVA, ovalbumin

We observed the histopathology of lung tissue by HE staining and noted that the alveolar wall in the OVA group was markedly thicker than that in the control group, with abundant inflammatory cell infiltration and glandular hyperplasia under the mucosa (Fig. 1A). Masson staining revealed that the content of collagen fibers in the lung parenchyma and airway was observably greater in the OVA group than in the control group (Fig. 1B). The levels of IgE and Th2 cytokines (IL-4, IL-5, and IL-13) in the BALF of the OVA group were notably greater than those in the BALF of the control group. However, the levels of Th1 cytokines (IFN-γ and IL-2) were markedly lower than those in the control group (Fig. 1C-E). RT‒qPCR revealed that DNMT1 expression was markedly lower and TSLP expression was markedly greater in the OVA group than in the control group (Fig. 1F‒G). Western blot analysis of DNMT1, TSLP and NF-κB pathway proteins revealed that DNMT1 expression was markedly lower and that TSLP, NF-κB p65 and p-IκBα expression was markedly greater in the OVA group than in the control group (Fig. 1H‒I). These results demonstrated that the mouse asthma model was successfully constructed, the IgE level and TSLP expression were increased, DNMT1 expression was decreased in asthmatic mice, the NF-κB signaling pathway was activated, and Th2 cell differentiation was increased in asthmatic mice.

### OS relieves asthmatic airway inflammation in mice

**Fig. 2:**
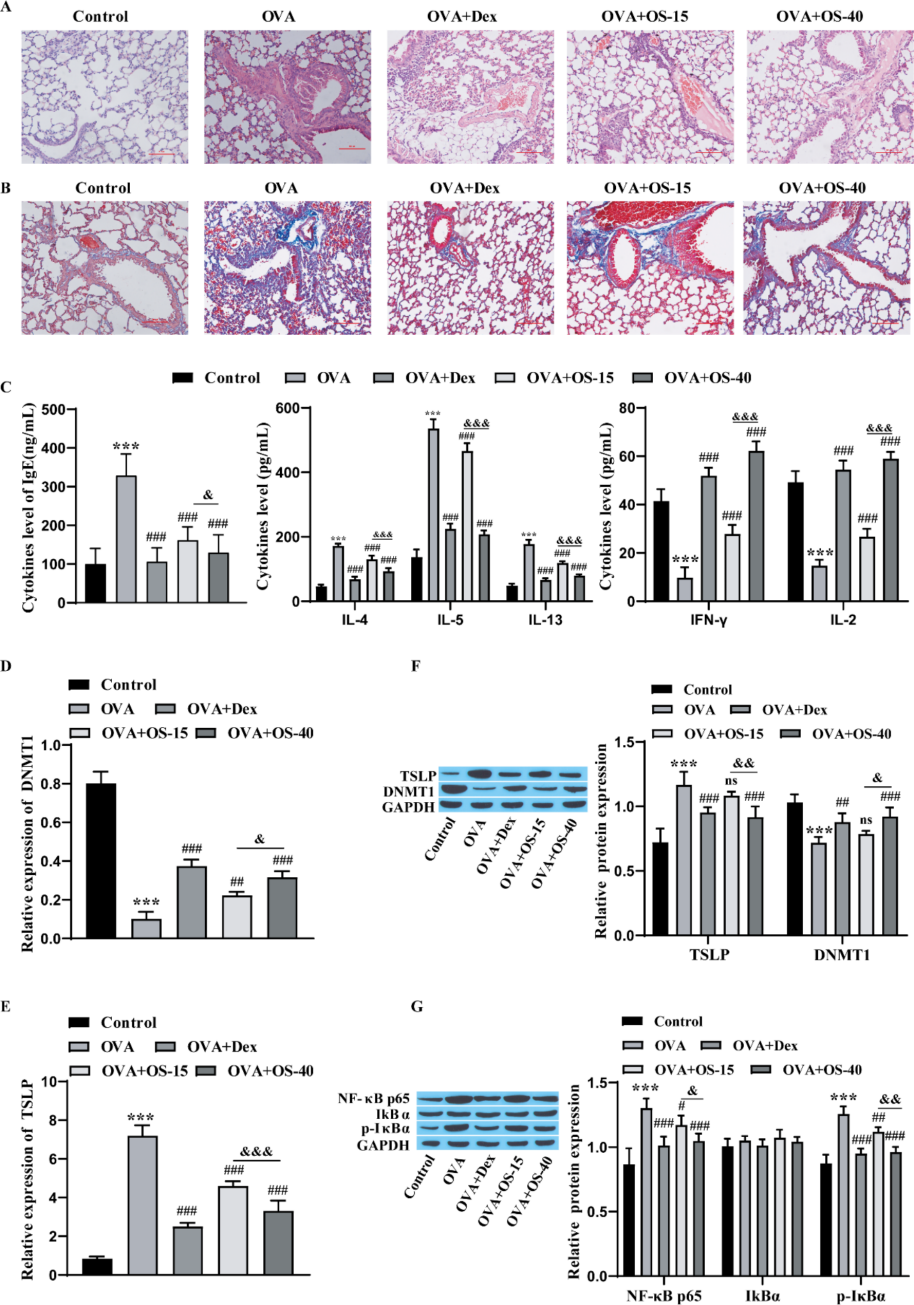
OS alleviates airway inflammation. **A**: HE staining was used for observing pathological alterations in lung tissue; scale bar = 100 μm. **B**: Collagen deposition was detected by Masson staining; scale bar = 100 μm. **C**: The IgE levels and the levels of Th2 and Th1 cytokines in BALF were detected by ELISA; **D**: DNMT1 levels were detected by RT‒qPCR; **E**: TSLP levels were detected by RT‒qPCR; **F**‒**G**: Western blot was used for detecting the protein levels of DNMT1, TSLP proteins and NF‒κB signaling pathway proteins. Animals were divided into 5 groups: control group (*n* = 5), OVA group (*n* = 5), OVA + Dex group (Dex, 1 mg/kg; *n* = 5), OVA + OS-15 group (*n* = 5), OVA + OS-40 group (*n* = 5). The experiments were repeated five times. ^***^P < 0.001 vs. Control; ^#^*P* < 0.05, ^##^*P* < 0.01, ^###^*P* < 0.001vs. OVA, ns: *P* > 0.05;^&^*P* < 0.05, ^&&^*P* < 0.01, ^&&&^*P* < 0.001 vs. OVA-15. “OVA + OS-40” means “OVA + 40 mg/kg OS”, and “OVA + OS-15” means “OVA + 15 mg/kg OS”. OVA, ovalbumin; Dex, dexamethasone; OS, osthole

To test the influence of OS on airway inflammation in asthmatic mice, we examined pathological alterations in lung tissue by HE staining. Compared with those of the control group, the alveolar walls of the OVA group were noticeably thicker, and there was abundant inflammatory cell infiltration and glandular hyperplasia under the mucosa. Both Dex and OS injection weakened the influence of OVA on lung pathology in mice, and the influence in the OVA + OS-40 group was greater than that in the OVA + OS-15 group (Fig. 2A). Masson staining revealed that the content of collagen fibers was observably greater in the OVA group than in the control group, Dex and OS treatment resulted in a prominent decrease in collagen fiber formation, and the degree of decrease in the OVA + OS-40 group was greater than that in the OVA + OS-15 group (Fig. 2B). Compared with those in the control group, the levels of IgE, IL-4, IL-5, and IL-13 in the OVA group were markedly elevated, while the levels of IFN-γ and IL-2 were notably decreased. Injection of both Dex and OS reversed the effects of OVA on each factor, and the degree of reversal was greater in the OVA + OS-40 group than in the OVA + OS-15 group (Fig. 2C). RT‒qPCR revealed that the level of DNMT1 was markedly lower and the level of TSLP was markedly greater in the OVA group than in the control group. Injection of both Dex and OS reversed the effect of OVA on DNMT1 and TSLP expression, and the degree of reversal was greater in the OVA + OS-40 group than in the OVA + OS-15 group (Fig. 2D-E). Western blot analysis revealed that DNMT1 expression was markedly lower and TSLP, NF-κB p65 and p-IκBα expression was markedly greater in the OVA group than in the control group. Both Dex and OS injection reversed the effects of OVA on DNMT1, TSLP, and NF-κB pathway protein levels, and the degree of reversal was greater in the OVA + OS-40 group than in the OVA + OS-15 group (Fig. 2F-G). These results suggest that OS can effectively reduce the levels of IgE, IL-4, IL-5, IL-13, TSLP, NF-κB p65 and p-IκBα, increase the level of DNMT1, and alleviate airway inflammation in asthmatic mice.

### OS alleviates LPS-induced apoptosis and inflammation in 16HBE cells and is associated with the NF-κB pathway

**Fig. 3:**
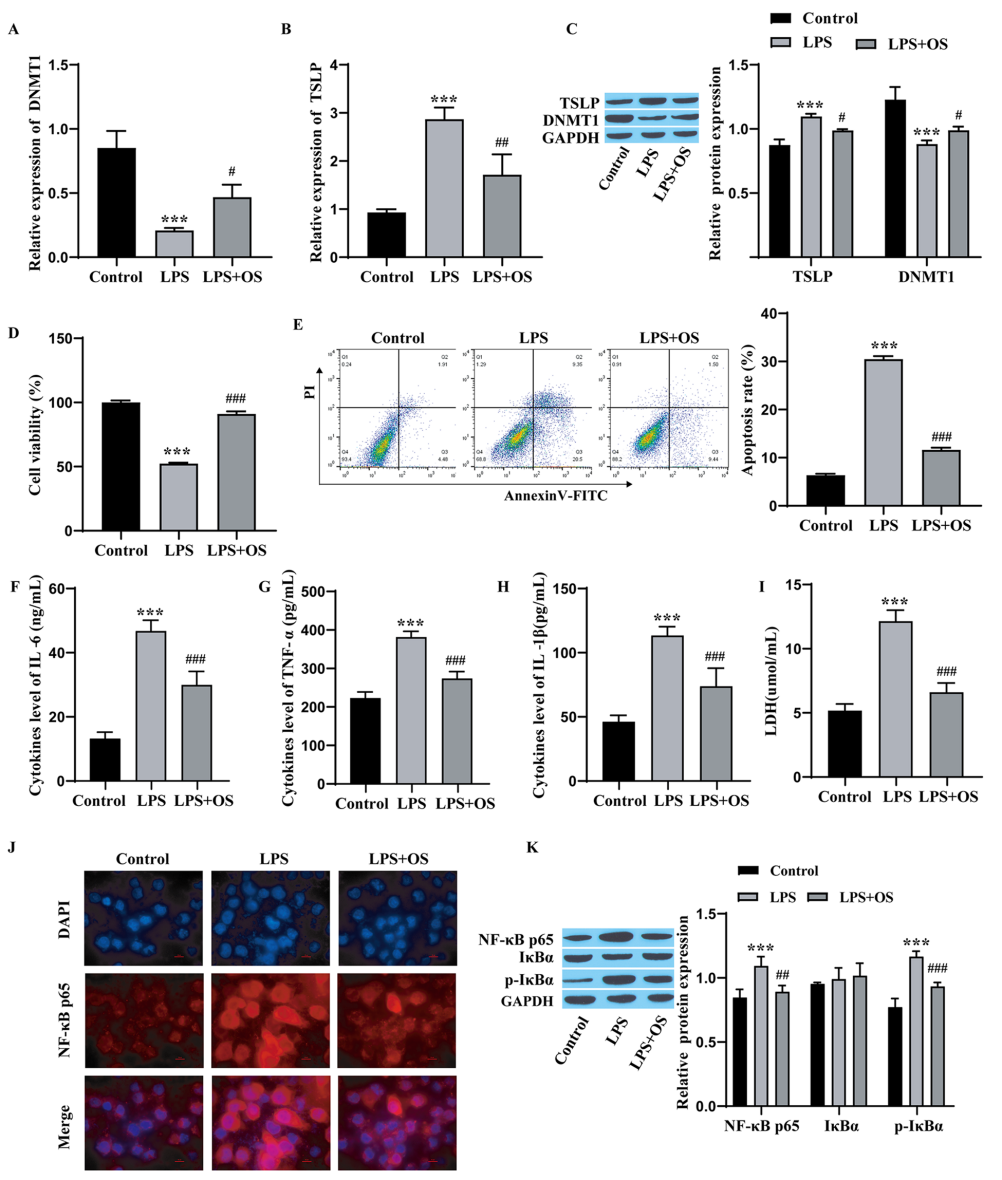
OS alleviates LPS-induced apoptosis and inflammation and is associated with the NF-κB pathway. **A**: RT‒qPCR for detecting DNMT1 levels; **B**: RT‒qPCR for detecting TSLP levels; **C**: Western blot for measuring the protein levels of DNMT1 and TSLP; **D**: CCK‒8 for assessing cell proliferation; **E**: Flow cytometry for assessing cell apoptosis; **F**‒**H**: ELISA for measuring the content of inflammatory factors; **I**: Kit for measuring LDH content; **J**: Immunofluorescence for detecting nuclear translocation of NF-κB; scale bar = 10 μm. **K**: Western blot for detecting the protein levels of the NF-κB pathway. 16HBE cells were divided into 3 groups: control group, LPS group (LPS, 1 mg/L), LPS + OS group (OS ,10 µg/mL). The experiments were repeated three times. ^***^P < 0.001 vs. Control; ^#^*P* < 0.05, ^##^*P* < 0.01, ^###^*P* < 0.001 vs. LPS. OS, osthole

To elucidate the effect of OS on LPS-induced 16HBE cell apoptosis and inflammation, we performed RT‒qPCR, and the results showed that the level of DNMT1 was markedly lower and the level of TSLP was markedly greater in the LPS group than in the control group. The addition of OS reversed these effects in the LPS group (Fig. 3A-B). Western blot analysis revealed that DNMT1 expression was notably lower and TSLP expression was prominently elevated in the LPS group compared to the control group. The effect in the LPS group was reversed after the addition of OS (Fig. 3C). CCK-8 detection of cell proliferation revealed that cell proliferation was noticeably lower in the LPS group than in the control group, and the addition of OS reversed the influence of LPS on cell proliferation (Fig. 3D). Flow cytometry revealed that apoptosis in the LPS group was observably elevated compared to that in the control group, and the influence of LPS on apoptosis was reversed after the addition of OS (Fig. 3E). ELISA revealed that the levels of IL-6, TNF-α and IL-1β were observably elevated in the LPS group compared with those in the control group, and the effect of LPS was reversed after OS was added (Fig. 3F-H). The LDH content was clearly greater in the LPS group than in the control group and was markedly lower in the LPS + OS group than in the LPS group (Fig. 3I). Immunofluorescence revealed that the nuclear translocation of NF-κB in the LPS group was observably elevated, and the effect of LPS was reversed after the addition of OS (Fig. 3J). Western blot analysis of NF-κB pathway proteins revealed that the levels of NF-κB p65 and p-IκBα were observably elevated in LPS group compared to those in the control group, and the effect of LPS was reversed after the addition of OS (Fig. 3K). These results show that OS can facilitate cell proliferation and DNMT1 expression; suppress apoptosis, NF-κB nuclear translocation, and IL-6, TNF-α, and IL-1β expression; reduce LDH levels; and alleviate LPS-induced apoptosis and inflammation in 16HBE cells.

### OS alleviates LPS-induced 16HBE cell apoptosis and inflammation by inhibiting the NF-κB pathway

**Fig. 4:**
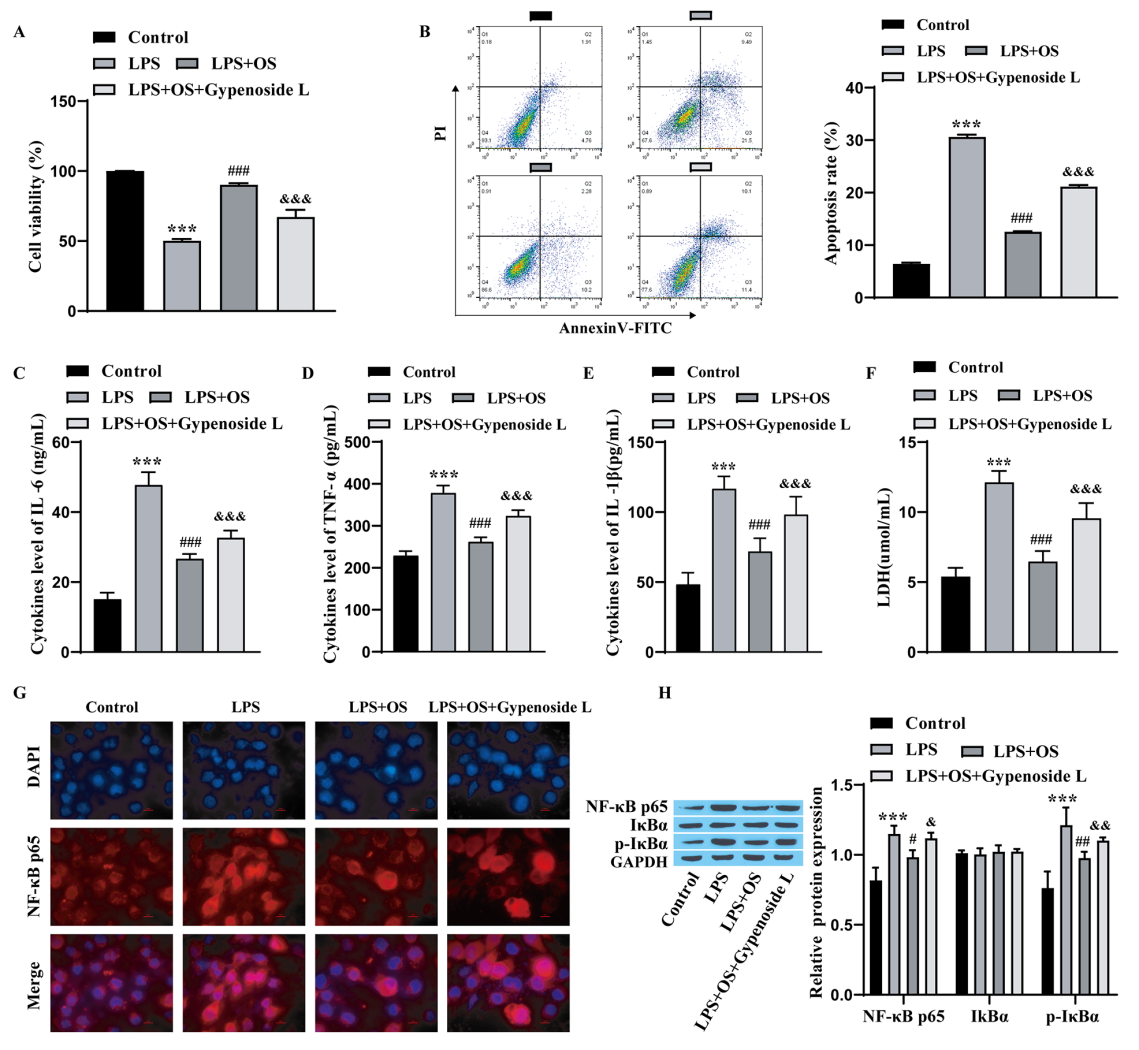
OS alleviates LPS-induced 16HBE cell apoptosis and inflammation by inhibiting the NF-κB pathway. **A**: CCK-8 assay for checking cell proliferation; **B**: Flow cytometry for checking apoptosis; **C**-**E**: ELISA for detecting the levels of inflammatory factors; **F**: kit for measuring LDH content; **G**: immunofluorescence for measuring the nuclear translocation of NF-κB; scale bar = 10 μm. **H**: Western blot for measuring the levels of NF-κB pathway proteins. 16HBE cells were divided into 4 groups: control group, LPS group (LPS, 1 mg/L), LPS + OS group (OS,10 µg/mL), LPS + OS + Gypenoside L group (Gypenoside L, 60 µg/mL). The experiments were repeated three times. ^***^P < 0.001 vs. Control; ^#^*P* < 0.05, ^##^*P* < 0.01, ^###^*P* < 0.001 vs. LPS; ^&^*P* < 0.05, ^&&^*P* < 0.01, ^&&&^*P* < 0.001 vs. LPS + OS. OS, osthole

The influence of OS on LPS-induced 16HBE cell apoptosis and inflammation through the inhibition of the NF-κB pathway was explored next. CCK-8 assays revealed that cell proliferation was lower in the LPS group than in the control group, and the addition of OS reversed the effect of LPS on proliferation, while the addition of gypenoside L (an activator of the NF-κB pathway) reversed the effect of the LPS + OS treatment on proliferation (Fig. 4A). Flow cytometry revealed that apoptosis was observably increased in the LPS group compared with that in the control group, decreased after the addition of OS compared to that in the LPS group, and increased after the addition of gypenoside L compared to that in the LPS + OS group (Fig. 4B). ELISA revealed that the levels of IL-6, TNF-α and IL-1β were prominently elevated in the LPS group compared to those in the control group, the influence of LPS was weakened after the addition of OS, and the effect in the LPS + OS group was suppressed after the addition of gypenoside L (Fig. 4C-E). LDH levels were greater in the LPS group than in the control group, markedly lower in the LPS + OS group than in the LPS group, and notably elevated in the LPS + OS + Gypenoside L group (Fig. 4F). Immunofluorescence revealed that the amount of nuclear NF-κB was observably elevated in the LPS group, the addition of OS weakened the effect of LPS, and the addition of gypenoside L reversed the effect in the LPS + OS group (Fig. 4G). Compared with those in the control group, NF-κB p65 and p-IκBα in the LPS group were prominently elevated, and the levels of NF-κB p65 and p-IκBα in the LPS group decreased after OS was added. The addition of gypenoside L reversed the effect of LPS + OS (Fig. 4H). These results showed that OS alleviates LPS-induced 16HBE cell apoptosis and inflammation by inhibiting the NF-κB pathway.

### OS inhibits Th2 differentiation and is associated with TSLP

**Fig. 5:**
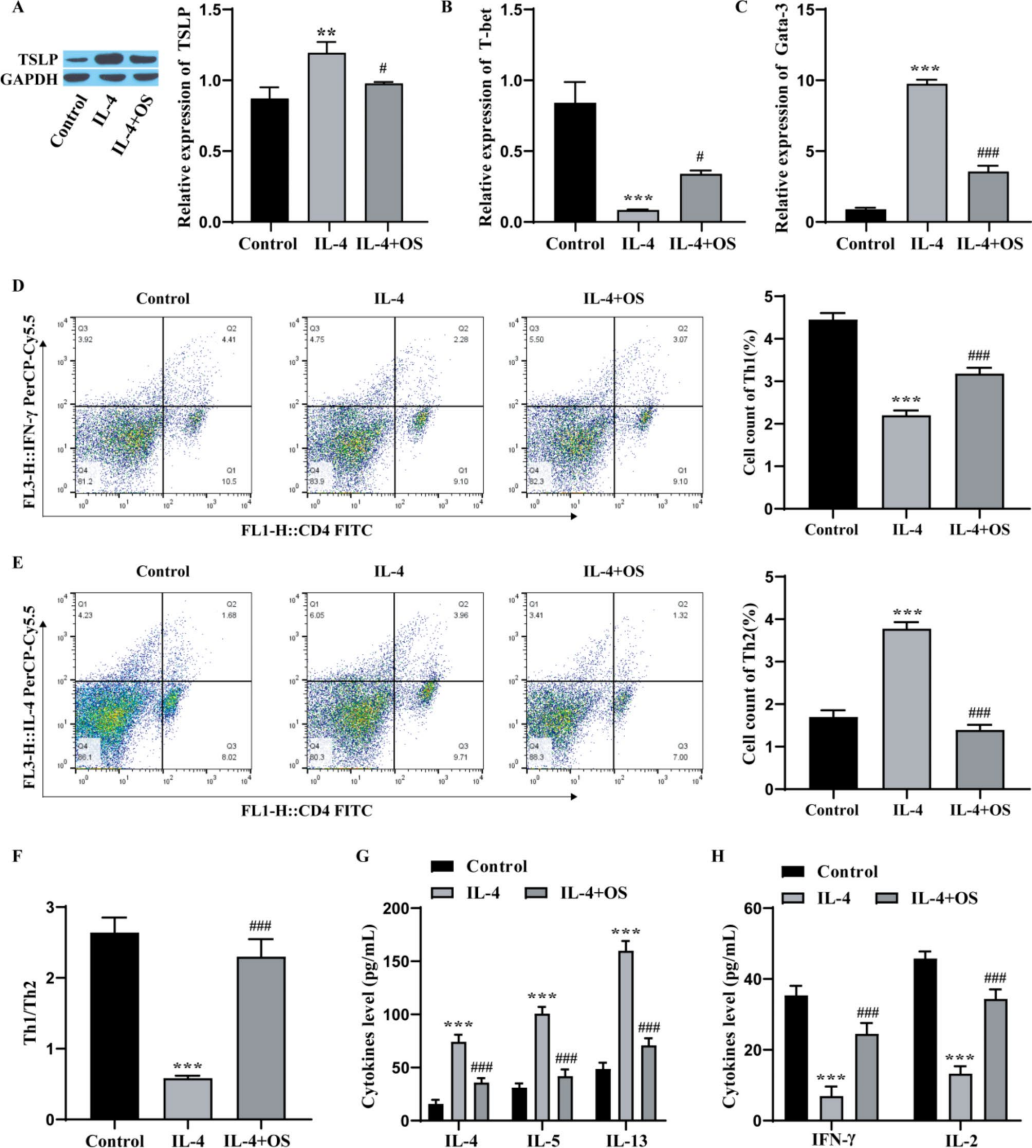
OS inhibits Th2 differentiation and is associated with TSLP. **A**: Western blot analysis of TSLP levels; **B**: RT‒qPCR analysis of T-bet levels; **C**: RT‒qPCR analysis of Gata-3 expression; **D**‒**F**: Flow cytometry analysis of the Th1/Th2 ratio; **G**: ELISA for detecting Th2 cytokine expression; **H**: ELISA for detecting Th1 cytokine levels. CD4 + T cells were divided into 3 groups: control group, IL-4 group (IL-4, 200U/mL), IL-4 + OS group (OS,10 µg/mL). The experiments were repeated three times. ^**^P < 0.01, ^***^P < 0.001 vs. Control; ^#^*P* < 0.05, ^###^*P* < 0.001 vs. IL-4. OS, osthole

To determine the influence of OS on the Th2 differentiation of CD4 + T cells, we carried out Western blotting, which revealed that OS inhibited IL-4-induced TSLP expression in CD4 + T cells (Fig. 5A). RT‒qPCR revealed that the level of T-bet was markedly lower and the level of Gata-3 was markedly greater in the IL-4 group than in the control group, and the effect in the IL-4 group was weakened after the addition of OS (Fig. 5B-C). Flow cytometry revealed that the number of CD4 + T cells that differentiated into Th1 cells in the IL-4 group was observably lower than that in the control group, while the number of CD4 + T cells that differentiated into Th1 cells in the OS group was observably greater than that in the IL-4 group. Moreover, the number of CD4 + T cells that differentiated into Th2 cells was prominently greater in the IL-4 group and prominently lower in the OS group than that in the IL-4 group, so the Th1/Th2 ratio was observably greater in the IL-4 + OS group than in the IL-4 group (Fig. 5D-F). ELISA revealed that the expression of IL-4, IL-5, and IL-13 was prominently elevated in the IL-4 group compared to the control group, while the levels of IFN-γ and IL-2 were noticeably decreased, and the addition of OS inhibited the effect of IL-4 (Fig. 5G-H). These results indicate that OS can restrain the levels of TSLP, Gata-3, IL-4, IL-5 and IL-13 and facilitate the expression of T-bet, IFN-γ and IL-2, thereby restraining Th2 differentiation.

### OS inhibits Th2 differentiation through TSLP

**Fig. 6:**
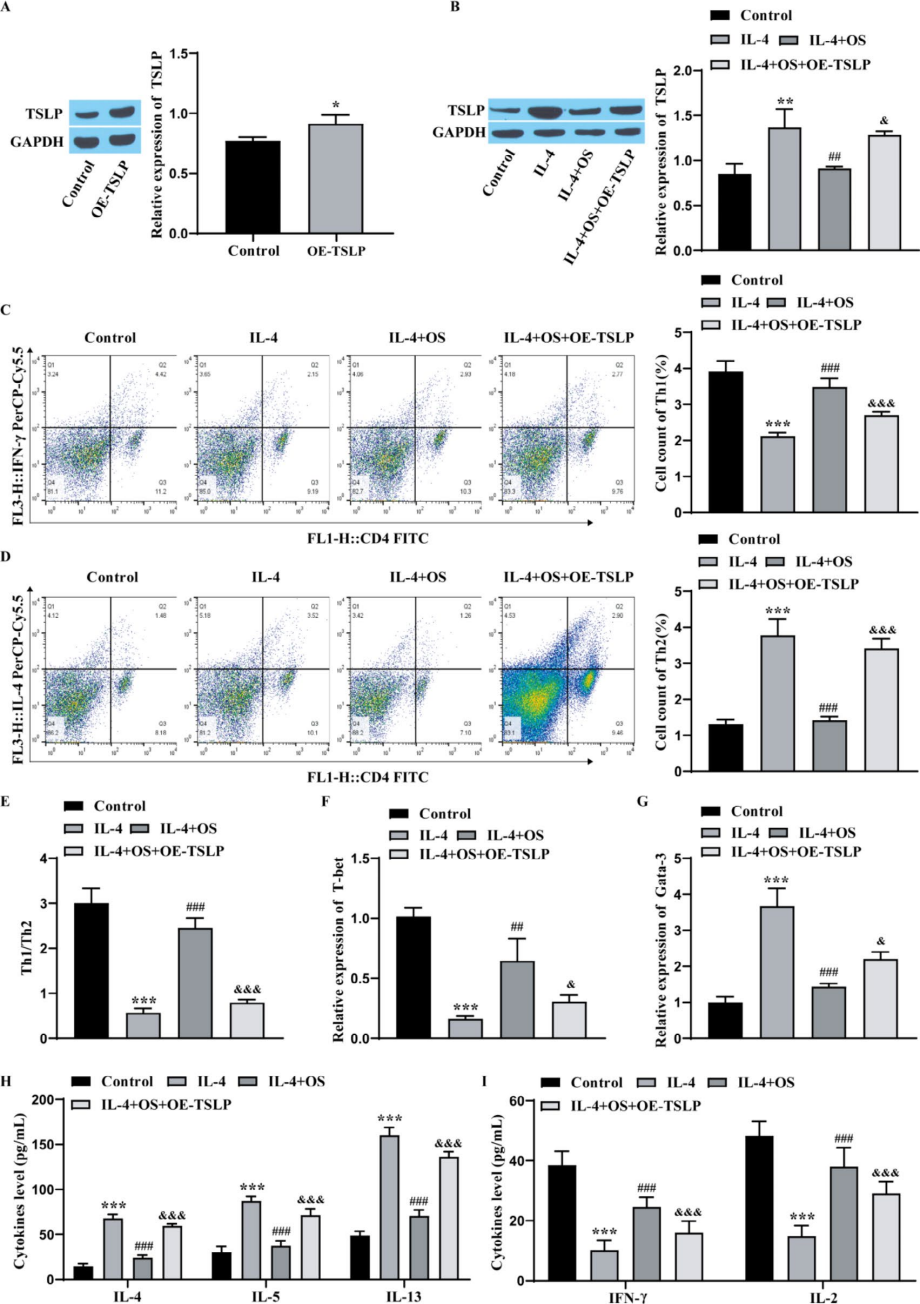
OS inhibits Th2 differentiation through TSLP. **A**: Western blot showing the transfection efficiency of OE-TSLP. **B**: Western blot showing the TSLP level. **C**-**E**: Flow cytometry showing the Th1/Th2 ratio. **F**: RT‒qPCR showing the T-bet level. **G**: RT‒qPCR showing the Gata-3 level. **H**: ELISA showing Th2 cytokine expression. **I**: ELISA showing Th1 cytokine expression. CD4 + T cells were divided into 4 groups: control group, IL-4 group (IL-4, 200U/mL), IL-4 + OS group (OS,10 µg/mL), IL-4 + OS + OE-TSLP group. The experiments were repeated three times. ^*^P < 0.05, ^**^P < 0.01, ^***^P < 0.001 vs. Control; ^##^*P* < 0.01, ^###^*P* < 0.001 vs. IL-4; ^&^*P* < 0.05, ^&&&^*P* < 0.001 vs. IL-4 + OS. OS, osthole

To confirm the influence of OS on the Th2 differentiation of CD4 + T cells through TSLP, we detected the expression of TSLP by Western blotting and found that TSLP expression was observably elevated in the OE-TSLP group compared with that in the control group, indicating successful transfection (Fig. 6A). Similarly, TSLP expression was markedly greater in the IL-4 group than in the control group, the effect of IL-4 was weakened after the addition of OS, and the effect in the IL-4 + OS group was reversed after the overexpression of TSLP (Fig. 6B). Flow cytometry revealed that the number of CD4 + T cells that differentiated into Th1 cells in the IL-4 group was markedly lower than that in the control group, the number of Th1 cells in the IL-4 + OS group was markedly greater than that in the IL-4 group, and the number of Th1 cells in the OE-TSLP group was lower than that in the IL-4 + OS group. Moreover, the number of CD4 + T cells that differentiated into Th2 cells in the IL-4 group was markedly greater than that in the control group, and after the addition of OS, the number of Th2 cells was significantly lower than that in the IL-4 group. Additionally, TSLP overexpression reversed the effects of OS, so the ratio of Th1/Th2 cells in the IL-4 + OS group was observably greater than that in the IL-4 group. The Th1/Th2 ratio was markedly lower in the IL-4 + OS + OE-TSLP group (Fig. 6C-E). RT‒qPCR revealed that the level of T-bet was markedly lower and that the level of Gata-3 was markedly greater in the IL-4 group than in the control group. The effect of IL-4 was weakened after adding OS. However, OE-TSLP reversed the influence of IL-4 + OS (Fig. 6F-G). Compared with those in the control group, the levels of IL-4, IL-5 and IL-13 in the IL-4 group were observably elevated, while the levels of IFN-γ and IL-2 were observably lessened, and the effect of IL-4 was inhibited after adding OS. Moreover, TSLP overexpression reversed the effect of IL-4 + OS (Fig. 6H-I). These results indicated that OS inhibited the Th2 differentiation of CD4 + T cells by inhibiting the expression of TSLP.

## Discussion

Asthma, characterized by wheezing, coughing and shortness of breath, is a chronic respiratory disease with a heterogeneous and multifactorial background, with clinical specificity in approximately 75% of patients [17]. Epidemiological studies have shown that asthma is more common in women than in men and is more common in children, especially boys [17]. The incidence of asthma continues to increase. OS is an artificial coumarin that has been widely applied to remedy multifarious diseases such as arthritis, hepatitis, atopic dermatitis, and allergic asthma [18]. Chiang [19] showed that OS treatment attenuates Th2-mediated allergic asthma by regulating the maturation and function of dendritic cells. Coincidentally, Yang [20] indicated that OS alleviates ovalbumin-induced lung inflammation by restraining IL-33/ST2 signaling in a mouse model of asthma. In addition, Dex, as an anti-inflammatory and immunosuppressant, can regulate the activity of Th1 and Th2 cells and participate in the treatment of asthma as well as allergic reactions [21]. In our research, to further probe the role of OS in the pathogenesis of asthma in mice, and according to the study of Li [3] et al., we treated model mice with 15 and 40 mg/kg OS and Dex, and found that the levels of TSLP, NF-κB p65 and p-IκBα were decreased and that the level of DNMT1 was elevated. After the injection of OS or Dex, the levels of IgE, IL-4, IL-5 and IL-13 were effectively reduced, the levels of IFN-γ and IL-2 were increased, the pathological state of the lung tissue was improved, and the airway inflammation of the asthmatic mice was alleviated. In addition, both 15 and 40 mg/kg OS had therapeutic effects on asthmatic mouse models, and 40 mg/kg OS had better therapeutic effects. These results suggest that OS has a similar function to Dex in inhibiting asthma and plays an important role in the regulation of immune balance.

NF-κB plays a major role in the expression of proinflammatory genes, including adhesion molecules, cytokines and chemokines; thus, the NF-κB pathway is considered a representative proinflammatory pathway [22]. Studies have shown that the NF-κB pathway is involved in the regulation of multifarious basic cellular processes, including the cellular immune response, cell proliferation, differentiation, apoptosis and inflammation [23]. Wang [24] reported that OS can decrease the inflammatory reaction in ovalbumin-induced allergic asthma by restraining the activation of NF-κB. Jin [25] reported that OS can effectively restrain the LPS-induced disruption of the NF-κB pathway in the lung tissue of acute lung injury (ALI) mice, thus inhibiting inflammation and tissue injury. In our study, we investigated the influence of OS on LPS-induced airway epithelial cell apoptosis and inflammation via the NF-κB pathway. The results showed that OS promoted cell proliferation and DNMT1 expression; inhibited apoptosis, NF-κB nuclear translocation and IL-6, TNF-α and IL-1β levels; and reduced LDH levels. Therefore, OS alleviates LPS-induced apoptosis and inflammation in airway epithelial cells. In addition, referring to previous studies [26], we treated cells with the NF-κB signaling pathway activator Gypenoside L (60 µg/mL) and found that the therapeutic effect of OS was weakened. These results suggest that NF-ĸB plays an important role in OS-mediated alleviation of the cellular inflammatory response.

TSLP is an epithelial-derived cytokine that attaches to the 4-helix bundle cytokine family and is a distal homolog of IL-7 [27]. Similar to IL-7, TSLP was shown to synergistically stimulate thymocyte proliferation and facilitate B-cell lymphopoiesis [28, 29]. Fu [7] reported that OS can reduce atopic dermatitis in mice by restraining TSLP produced by keratinocytes and that OS can also inhibit the Th2 cell response. Moreover, Furue [15] showed that the release of a large amount of TSLP can initiate Th2 and Th22 immune responses. Roan [30] showed that TSLP produced by barrier surface epithelial cells activated dendritic cells (DCs) expressing TSLP receptor (TSLPR) to induce the production of functional Th2 cells. In addition, studies have shown that TSLP is an important regulator of DC function and can activate DCs to induce CD4 + T-cell proliferation and Th2 cell differentiation and subsequently produce inflammatory factors (IL-4, IL-5, and IL-13) [31]. This finding suggested that TSLP can regulate DC cells to influence the Th2-mediated inflammatory response. In this study, we investigated the effect of OS on the Th2 differentiation of CD4 + T cells through TSLP. The results showed that OS could restrain the levels of TSLP, Gata-3, IL-4, IL-5 and IL-13; increase the expression of T-bet, IFN-γ and IL-2; and subsequently inhibit the Th2 differentiation of CD4 + T cells. However, TSLP overexpression weakens OS, promotes Th2 differentiation of CD4 + T cells, and subsequently releases inflammatory factors.

## Conclusion

This study revealed that OS inhibited Th2 differentiation by suppressing the TSLP and NF-κB pathways through the inhibition of NF-κB nuclear translocation, thereby reducing asthma-induced airway epithelial cell apoptosis and inflammation and alleviating asthma. In this study, we only detected this phenomenon through cell and mouse experiments. Further research is needed to determine the clinical safety and efficacy.

## Electronic supplementary material

Below is the link to the electronic supplementary material.

Supplementary Material 1

## Data Availability

The datasets used and analyzed during the current study are available from the corresponding author upon reasonable request.
