# Supplementary material for: Osthole attenuates asthma-induced airway epithelial cell apoptosis and inflammation by suppressing TSLP/NF-κB-mediated inhibition of Th2 differentiation
Source: Allergy Asthma Clin Immunol. 2024 Sep 27;20:51. doi: 10.1186/s13223-024-00913-8 (PMC11438018; doi:10.1186/s13223-024-00913-8)
Supplement: Supplementary file 1 — Supplementary Material 1 [file 13223_2024_913_MOESM1_ESM.pdf]

Editing Certificate

This document certifies that the manuscript

Osthole by suppressing TSLP/NF-κB inhibition of Th2 differentiation attenuates asthma-induced airway epithelial cell apoptosis and inflammation

prepared by the authors

Yanli Li1, Yushan Zhou1, Liqiong Liu1, Yunfeng Yang1, Yanhong Liu1, Dailing Yan1, Juyan Chen1, Yi Xiao1*

was edited for proper English language, grammar, punctuation, spelling, and overall style by one or more of the highly qualified native English speaking editors at AJE.

This certificate was issued on **March 11, 2024** and may be verified on the AJE website using the verification code **6F89-BEB5-6C42-0C9E-F317** .

Neither the research content nor the authors' intentions were altered in any way during the editing process. Documents receiving this certification should be English-ready for publication; however, the author has the ability to accept or reject our suggestions and changes. To verify the final AJE edited version, please visit our verification page at aje.com/certificate. If you have any questions or concerns about this edited document, please contact AJE at support@aje.com.

AJE provides a range of editing, translation, and manuscript services for researchers and publishers around the world.
For more information about our company, services, and partner discounts, please visit aje.com.
